# Collective Violence against Health Workers in the Context of the COVID-19 Pandemic

**DOI:** 10.3390/nursrep13020079

**Published:** 2023-06-14

**Authors:** Davina Jacobi, Tobias Ide

**Affiliations:** 1 Discipline Area of Nursing, IUBH International University, 53604 Bad Honnef, Germany; davina.jacobi@gmx.de; 2 Center of Biosecurity and OneHealth, Murdoch University, Murdoch, WA 6150, Australia

**Keywords:** attack, conflict, COVID-19, health worker, public health, nurse, pandemic, politics, violence

## Abstract

Concerns about violence against nurses and other medical personnel have increased during the COVID-19 pandemic. However, as of yet, limited systematic knowledge of such violence is available. Addressing this gap, we analyse the geographical distribution of, motivations behind, and contexts of collective attacks against health workers in the context of the COVID-19 pandemic. To do so, we systematically recorded and coded attack events worldwide from 1 March 2020 to 31 December 2021. We identify high-risk countries, attack characteristics, and the socio-economic contexts in which attacks tend to occur. Our results show that opposition against public health measures (28.5%), fears of infection (22.3%), and supposed lack of care (20.6%) were the most common reasons for attacks. Most attacks occurred in facilities (often related to a supposed lack of care) or while health workers were on duty in a public place (often due to opposition to public health measures). However, 17.9% of all attacks took place in off-duty settings. Democratic countries with high vaccination rates and strong health systems were relatively safe for nurses and doctors. Distrust in the skills of health workers and the science underlying health interventions is a major driver of collective attack risks and should be addressed before it turns violent. This study was not registered.

## 1. Introduction

Violence against health workers has emerged as a major area of concern during the COVID-19 pandemic. Already a few months after declaring a pandemic, the World Health Organization [1] called for more actions “to protect health workers from violence” and identified the safety of nurses, doctors, and paramedics as a major area of concern. Likewise, a comment published in The Lancet around the same time highlighted that “attacks against health-care personnel must stop, especially as the world fights COVID-19” [2] (p. 1743). Lawmakers in several countries—including Australia, Belgium, China, India, Sudan, and the USA—recently introduced tougher penalties for offenses against medical personnel. By doing so, they responded to increased rates of violence since the start of the pandemic [3] and to evidence that health workers perform key roles in responding to and dealing with pandemics [4]. Examples of COVID-19-related violence against health workers include assailants throwing chlorine at a nurse due to fears of infection in the Philippines, a care worker being attacked by anti-lockdown protestors in Austria, and policemen in Sudan severely beating a doctor requesting the evacuation of a hospital due to the spread of COVID-19 [5,6].

Harassment of health workers is not a new phenomenon. A systematic review of workplace violence published in 2019, for instance, found that more than 24% of all health workers experienced physical attacks in the previous 12 months [7]. Other cross-national analyses reported that 36% of nurses and 65% of emergency medical personnel had been physically attacked at least once during their working life [8,9]. Studies also revealed considerable levels of physical violence against health workers in individual countries, including China [10], Germany [11], Pakistan [12], and the USA [13]. Healthcare workers employed in emergency and mental health wards [14] and living in armed conflict-affected countries [15] face particularly high attack risks. All studies found that the frequency of verbal threats and psychological violence is higher than the frequency of physical attacks, even though the latter can be more devastating for the victims.

Past research has established that attacks against health workers have severe consequences, including physical injuries, psychological trauma and stress, more people leaving the healthcare sector, and reduced abilities of health workers to perform crucial tasks [8,16]. Consequentially, it is highly important to study not just the prevalence of such forms of violence but also when, by whom, and in which contexts such attacks are conducted.

Despite prominent calls to gather new evidence and high demand for such information [2], our knowledge of violence against health workers in the context of the COVID-19 pandemic is still limited. Dye et al. [17] surveyed 173 countries and found that already in April 2020, more than 10% of the health workforce in North America, South Asia, and Sub-Saharan Africa reported COVID-19-related (verbal and physical) harassment. Other studies analysed pandemic-related violence incidents in particular world regions, such as Pakistan [18] or Latin America [19], or discuss the topic in general, drawing on anecdotal evidence to make recommendations for decision-makers [13,20]. 

So far, evidence on the contexts and motivations of attacks is largely absent, despite its relevance for designing preventive measures and policies. Likewise, no research has focussed specifically on collective violence, which involves several perpetrators physically attacking, restricting, or threatening health workers. Compared to violence by individuals, such collective attacks result in a higher risk of injury, lower chances to escape, and more intense psychological stress [21].

This study addresses the knowledge gaps identified above. It systematically collects and analyses incidents of collective violence against nurses, doctors, and paramedics worldwide in the context of the COVID-19 pandemic for the period 1 March 2020 to 31 December 2021. By doing so, we contribute to existing knowledge in three main ways. To start with, we conduct the first study on collective (physical) violence against health workers. Second, using an approach grounded in peace and conflict studies [22], we focus on reported events of violence rather than on survey data. Third, this allows us to disentangle the reasons for and contexts of the attacks.

## 2. Materials and Methods

### 2.1. Definitions and Scope of the Analysis

For the purpose of this study, we define collective violent attacks against health workers as efforts by at least two perpetrators to physically harm or restrict persons employed in the healthcare sector, such as nurses, doctors, and paramedics. This includes incidents where the health worker is able to flee from the attack or where imminent threats of physical violence are used to enforce a certain behaviour of the health worker, such as providing treatment or staying at home [6,17]. The actions of the perpetrators need to be linked to the professional status or actions of health workers [9]. For instance, we do not include anti-lockdown protests during which a nurse has been injured unless the nurse was specifically targeted due to his/her profession. Our study also does not cover attacks solely targeting health infrastructure rather than personnel (e.g., arson in hospitals under construction). An attack is a discrete event that happens during a particular time at a specific place, no matter how many perpetrators or targets are involved.

Geographically, our study is global in nature, focussing on all countries worldwide, even though for some regions and countries, limited data are available (see below for a detailed discussion of data limitations).

### 2.2. Sample and Data

Data on attacks against health workers came from two sources: (1) the Armed Conflict Location and Event Data Project (ACLED), which surveys collective violence and protest events worldwide based on media reports and expert assessments [5,23], and (2) the Health Care at Risk (HCR) database, which collects information on violence and threats in health care contexts based on reports from various international governmental and non-governmental organisations [6] Both datasets were downloaded on 19 February 2022).

**Figure 1:**
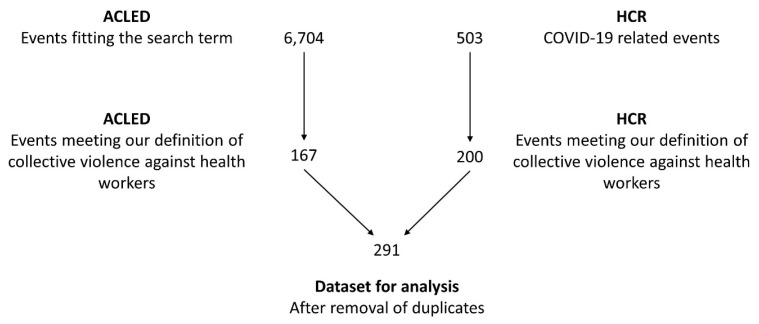
Visualisation of the data collection and filtering process.

For ACLED, we used the Boolean search term “covid OR corona AND nurse OR doctor OR physician OR paramedic OR health worker OR healthworker OR healthcare worker OR healthcare staff OR hospital” to identify relevant events. For HCR, we considered all attacks classified as COVID-19-related in the dataset. Both authors manually read and coded each entry to check whether it fell under the definition of a collective, violent attack provided above, to make sure the event was related to the COVID-19 pandemic, to remove duplicates, and to register additional information on the attack. Any disagreements between the authors about the inclusion of an event into the dataset were resolved by means of discussion until consensus was achieved (see Figure 1 for an overview).

In order to address the research gaps and goals outlined in the introduction, we collected information on the context in which the attack occurred, particularly whether the health worker was attacked on duty or off duty. Attacks while on duty were further classified as happening in a facility (e.g., a hospital or vaccination clinic) or in a public place (e.g., when driving an ambulance or conducting COVID-19 screenings). Off-duty incidents could also either occur in a public place (e.g., on a bus or a street) or at (or very close to) home. 

We further distinguished between five major motivations behind an attack: supposed lack of care (e.g., when relatives accused the health worker of being responsible for the death of a family member), opposition to public health measures (e.g., quarantine, COVID screening), fears of health workers infecting others (because they work in setting where COVID-19 is prevalent), demand for vaccinations (e.g., by people not eligible to receive them), and opposition to vaccinations (e.g., attacks against vaccination clinics/workers). Both authors coded all events independently of each other and resolved disagreements via discussion to produce a harmonised dataset.

We conceive the dataset as a major innovation of our research. In contrast to existing studies, it allows us to include information on the contexts of and motivations for collective attacks in our analysis on a global scale. However, as with other (e.g., self-reported or survey-based) data, there are also biases and shortcomings. Specifically, as attack reports come from media reports (ACLED) and governmental and professional associations (HCR), attacks in autocratic countries with state-controlled media and professional associations are likely to be underreported. Media reporting of events also follow thematic trends, implying that during time periods when public attention is on other issues (e.g., vaccination development, new variants of COVID-19), attacks are also underreported [24].

### 2.3. Data Analysis

Once the dataset was compiled and coded, we counted the number of attacks per country. As these numbers could be misleading due to the very different population sizes, we also created a risk-of-attack indicator by determining the number of attacks per ten million inhabitants based on 2020 population data from the World Bank [25]. 

Afterward, we analysed how the average risk-of-attack indicator varied along various socio-economic characteristics of country groups. These characteristics included: the level of human development as indicated by the United Nations Development Programme’s [26] Human Development Index (HDI), the number of physicians per 1000 inhabitants as recorded by the World Bank [25], the level of democracy provided by the polity2 value of the polity V project [27], and the percentage of the population double-vaccinated against COVID-19 on 31 December 2021 according to Ritchie et al. [28]. By analysing the economic, political, and demographic characteristics of countries with high or low attack risks, we intended to shed further light on the contexts in which attacks occur and potential preventive measures.

**Figure 2:**
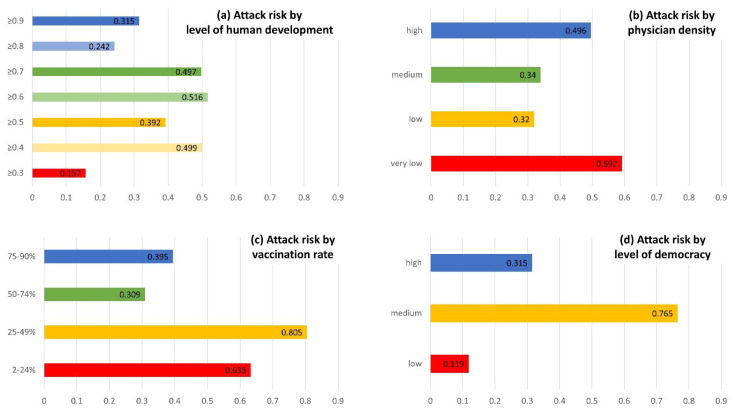
Risk of collective, COVID-19-related violent attacks against different socio-economic country groupings (see Section 2 and Supplementary Material for further information).

Given the small-N (data for 164 countries were available) and because the key drivers of collective attacks are highly localised and situation-specific, regression analyses at the country level were unlikely to yield significant results. In this article, we, therefore, report robust patterns of attack risk clustered by the socio-economic country groupings (see Figure 2). The results are in line with Pearson’s correlation coefficients which we calculated for the relevant variables, even though these coefficients are not statistically significant (for the reasons discussed above). All correlation coefficients are reported in the Supplementary Material.

The Supplementary Material also provides the full dataset, including all events, their coding, and the economic, political, and demographic data used in the analysis.

## 3. Results

Altogether, we detected 291 instances of COVID-19-related, collective violent attacks against health workers. In this context, one should consider that an estimated 60–80% of such attacks go unreported, even when drawing on official statistics in open and democratic societies [13,29].

**Figure 3:**
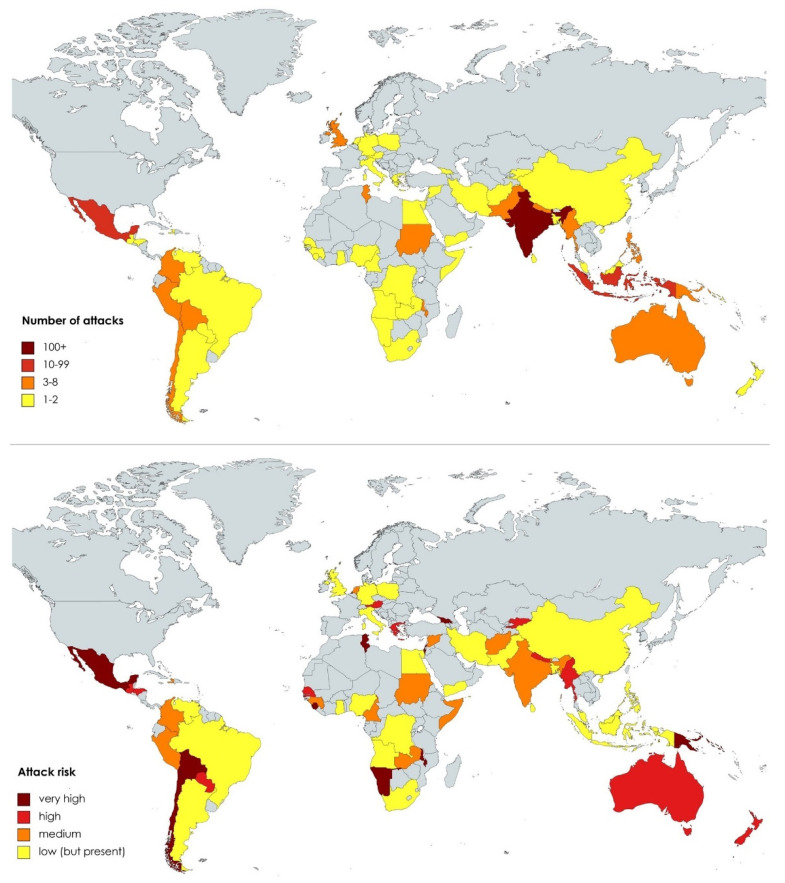
Number of attacks against health workers (upper map) and population-adjusted attack risk (lower map).

The upper map in Figure 3 shows the number of attacks per country. Most attacks are reported from India (127), Mexico (33), Indonesia (13), Tunisia (8), and Myanmar (8). However, these numbers can be misleading because they do not account for different population sizes. The population-adjusted attack risk indicator (Figure 3, lower map) shows that the Solomon Islands (an outlier with 29.18 attacks/10 million people), Tunisia (6.77), Bolivia (5.14), Namibia (3.94), Malawi (3.66), and Papua New Guinea (3.35) were the most dangerous countries for health workers in terms of pandemic-related, collective violence. Latin America and the southern parts of Asia have most countries with a high prevalence of attacks. Surprisingly, large countries with well-documented incidents of individual attacks against health workers, such as the USA or Russia, did not register any collective attack [13], indicating potential underreporting issues in the dataset.

**Figure 4:**
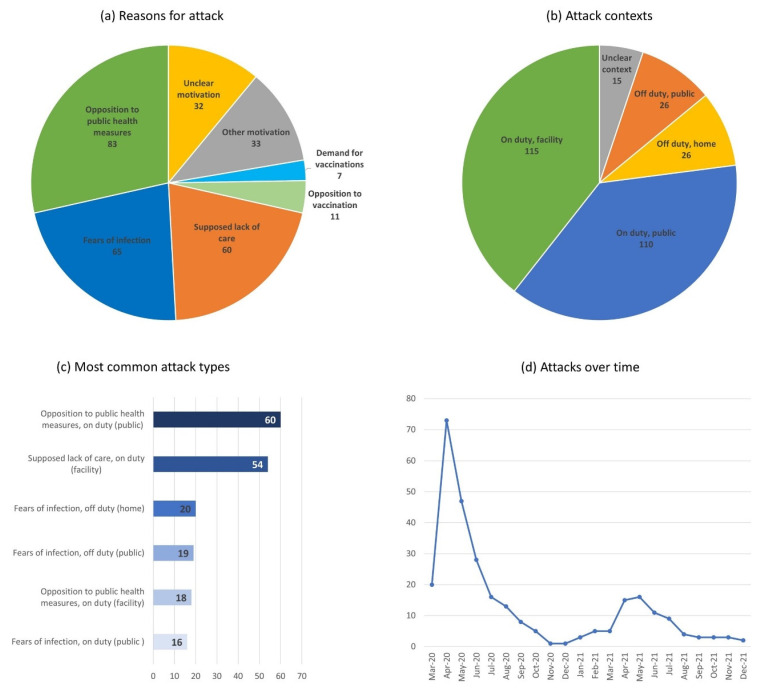
COVID-19-related, collective violent attacks against health workers (**a**) by reason, (**b**) by context, (**c**) by attack type, and (**d**) over time.

Figure 4 provides an overview of the attack motivations, contexts, and types (see Section 2 for a discussion of data sources and the Supplementary Material for the complete data). As indicated by panel a (top left corner of Figure 4), opposition against public health measures, such as quarantine or COVID-19 screening, was the most common reason for an attack (28.5%), followed by fears that the health worker would infect others with COVID-19 (22.3%), and a supposed lack of care for patients who suffered or died from COVID-19 (20.6%). Attacks in the context of vaccination campaigns only account for 3.8% of all incidents but for 15.1% of all attacks recorded since February 2021 (when vaccinations became widely available). Attacks in opposition to vaccinations were slightly more likely than attacks by perpetrators demanding a vaccination.

Panel b (top right corner) of Figure 4 illustrates how common different attack contexts are. The large majority of collective attacks against health workers occurred while they were on duty, either in a facility (39.4%) or when providing health services in a public place (37.7%). At the same time, a relevant amount (17.9%) of COVID-19-related attacks took place while health workers were off duty, either in a public place (8.9%) or at home (8.9%).

Figure 4, panel c (bottom left corner) pairs the attack motives and contexts to identify the most common attack types. Most widespread were attacks motivated by opposition to public health measures while the worker was on duty in a public place (20.6%) and attacks motivated by a supposed lack of care at the workplace (18.6%). This is plausible given that health workers often carry out or support public health measures in public, while patients and their relatives interact with health workers mostly in hospital settings. However, attacks motivated by opposition to public health measures were also not uncommon at the workplace (6.2%). Already ranking third and fourth are attacks due to fears of infection while the health worker was off duty at home (6.9%) and in a public place (6.5%). Table 1 contains a brief narrative example for each of the six most common attack types.

Figure 4, panel d (bottom right corner), visualises the temporal patterns of attacks. Collective violence against health workers was prevalent in the first months of the pandemic but declined rapidly from May 2020 onwards. With the exception of April and May 2021 (corresponding with a strong rise in global infections), the number of monthly incidents remains below 15.

Figure 2 displays the average attacks risk for different country groups based on their political-economic characteristics (excluding the Solomon Islands as an outlier case). According to panel a (top left corner of Figure 2), there is no general association between the level of human development and attack risks, even though the most developed countries face slightly fewer attacks. There are differences between the average collective attack risk index when clustering countries according to their number of physicians per 1000 inhabitants (panel b, top right corner of Figure 2). Specifically, the attack risk is higher in countries with very high numbers of physicians and with very low numbers of physicians. However, the differences between the country groups are relatively small. Panel c (bottom left corner of Figure 2) illustrates that countries with high two-dose vaccination rates (≥50%) have, on average, much lower attack risks. Finally, average risks for collective attacks on health workers seem to be lowest in autocratic states, relatively low in strong democracies, and very high in countries with a medium level of democracy (panel d, bottom right corner of Figure 2).

## 4. Discussion

Our results demonstrate that collective violence against health workers is a global challenge. As Figure 3 (lower map) and Figure 2 (panel a, top left corner) demonstrate, collective COVID-19-related attacks against health workers are reported in rich and poor countries, and there is no significant correlation with the level of human development. This finding challenges widespread perceptions of Mexico and particularly India as outstandingly high-risk countries [19,30]. In fact, India is only ranked 23rd in terms of attack risk, behind countries usually considered rather safe, such as New Zealand (12) or Austria (21). Our results are in line with other studies demonstrating increasing amounts of violence against health workers during other pandemics, such as Ebola or Cholera [31].

With opposition to COVID-19-related health measures being the most common motivation for attacks (28.5%), we see that health workers often function as scapegoats. They are attacked because the perpetrators blame them for political decisions that the health workers did not make but “merely” follow and implement. Examples of this include collective attacks on health workers conducting COVID-19 screenings or working in vaccination clinics.

Our finding that 77.3% of all attacks happen on duty is well in line with earlier studies highlighting the risks of workplace violence in the health sector [7,17]. Yet, more than one in seven attacks (17.9%) occurs off duty. This suggests that during the pandemic, shopping centres, public transport, and even their own home have not been safe spaces for health workers. For this reason, many Mexican and Philippine nurses stopped wearing their uniforms when commuting to or from work in April 2020 [32].

Taken together, these findings suggest that frequently suggested safety measures, such as security buttons and access limitations in hospitals, can help to reduce collective violence against health workers [13]. However, with 46.7% of all attacks happening in public places and 17.9% off duty, such measures alone are insufficient to guarantee the safety of health workers (Figure 4, panel c, bottom left corner). Public education campaigns highlighting the skills and importance of health workers or portraying them as life savers rather than disease spreaders could also address violent attacks that occur in public places and/or off duty.

The declining trend of attacks over time can be attributed to two (mutually not exclusive) reasons. First, attacks declined after initial uncertainty about COVID-19 and its health consequences lessened. With the increasing availability of vaccinations, treatments, and protective equipment and more information on the infection chains of COVID-19, people could have been less concerned about being infected by health workers living in the same street. In line with this, fears of infection only accounted for four attacks in 2021. Second, while the topic of violence against health workers featured prominently in media reports at the beginning of the pandemic [13], attention shifted to other issues, such as vaccinations and new variants over time [33]. Therefore, underreporting of events of collective violence against health workers has likely increased from mid-2020 onwards. More systematic data collection is a key task for future research in order to produce more comprehensive analyses and develop recommendations for reducing collective violence against health workers.

There is a weak association between the number of physicians per 1000 inhabitants and the risk of COVID-19-related collective violence against health workers (Figure 2, panel b, top right corner). Specifically, the attack risk is higher in countries with very high numbers of physicians (presumably because more interactions with health workers result in more attacks) and with very low numbers of physicians (likely sparked by general dissatisfaction with the state of the health system). In countries with medium to low numbers of physicians, doctors are less available as targets of attacks, yet the health system is still relatively functional. However, the differences between the country groups are rather small.

Furthermore, we found that attacks occurred more frequently in countries with low vaccination rates. Three factors are likely to explain this pattern: First, vaccinations protect against getting infected with and suffering from severe symptoms of COVID-19, hence reducing anxiety about infection risks and inadequate care and the need for public health measures (all of which are major reasons behind attacks). Second, the ability to quickly deliver vaccinations to a large part of the population increases the reputation of the entire health system, including health workers. Third, high vaccination rates indicate lower levels of distrust in the science behind COVID-19. Such distrust is a major driver of attacks opposing public health measures [34], such as violence against the personnel of vaccination clinics. All too often, health workers (who sometimes implement but never decide on public health measures) are used as scapegoats, while attacks are an act of resistance against these measures. 

Given that highly developed democratic countries usually have well-developed health systems, strong public institutions, and a culture of non-violent conflict resolution [35], it should come as no surprise that they experience fewer attacks on average (Figure 2, panel d, bottom right corner). The low attack risk in autocratic states, by contrast, is very likely driven by reporting bias: censored media, government departments with a strong political agenda, and highly restricted non-government organisations are unlikely to report collective violence against health workers. This is an important shortcoming of the dataset used here, but also of other datasets that include non-democratic countries. However, in autocratic regimes, opportunities for protests and public demonstrations are also limited, and several attacks against health workers have occurred in the context of such protests. This might be another reason why attack numbers in autocratic countries tend to be low. Countries with medium levels of democracy, by contrast, lack the institutional context that can prevent violence yet have sufficient independent media and non-governmental organisations to report attacks against health workers.

This study comes with a number of limitations. The datasets we use are based on the reports of media, governments, and non-governmental organisations (such as professional medical associations). This means that when press freedom is limited, when associations face strict state control, or when media attention shifts from one topic (e.g., violence against nurses) to another (e.g., new virus variants), a bias enters our data. Furthermore, collective attacks against health workers are often driven by highly localised factors (e.g., the presence of bystanders or security measures in hospitals) on which no cross-case data are available and which we can consequently not include in our analysis. Finally, given the lack of information on localised factors and the small size of our sample, robust (multivariate) statistical analyses could not be applied.

## 5. Conclusions

We conducted the first global, event-based study on collective violence against health workers in the context of the COVID-19 pandemic. Our results make clear that despite prevalent underreporting, such attacks against nurses, doctors, and paramedics are a problem affecting all world regions. Singling out particular countries, such as India or Mexico, is not justified. The prevalence of collective violence is concerning, given the key role of health professionals in delivering health care, dealing with pandemics, and achieving sustainable development [36]. 

Overall, democratic countries with high vaccination rates and strong health systems experience, on average fewer attacks. Most attacks are motivated by a perceived lack of adequate care (20.6%), fears of COVID-19 infection (22.3%), and particularly opposition to public health measures (28.5%). Health workers are often scapegoats and available targets for people aggrieved about such measures. While 77.1% of all attacks occur on duty, health workers also face considerable insecurity off duty (17.9%) and in public places (46.7%). 

Taken together, this indicates that workplace-related measures, such as security buttons, are helpful in reducing collective (and individual) violence against health workers. However, workplace-related measures need to be complemented by investments in health systems and public education campaigns. Distrust into the skills of health workers and the science underlying health interventions is a major driver of collective attack risks. Consequentially, they should be addressed by education and public relations activities before they turn violent. Our study identified various pathways through which insufficient health systems can result in more collective violence against health workers, such as the low ability to deliver vaccines and insufficient capacities to treat patients. Investments into health systems would pay off twice, first by improving the systems itself and second by reducing attacks on doctors, nurses, and paramedics (which further strengthens health systems).

More research is also necessary on the situational contexts making collective violence against health workers more likely in order to tailor safety strategies to the respective local context. Future work should collect more fine-grained data on collective attacks to learn more about their very local and situational contexts. Surveys with health workers and interviews with perpetrators of attacks, for instance, could provide valuable insights on why collective, violent attacks occur and how they can be prevented.

## Figures and Tables

**Table 1:** Most common attack types with examples (These examples were extracted from the ACLED and HCR databases. See Section 2 for details and the Supplementary Material for full information on these events)).

Attack Type	Example
Opposition to public health measures, on duty (public)	30 March 2020, Jerusalem, Israel: A group of ultra-orthodox people threw rocks at health workers conducting COVID-19 tests.
Supposed lack of care on duty (facility)	5 April 2021, Vadodara, India: More than 60 people vandalised a hospital and attacked staff members after a woman admitted due to COVID-19 died in the hospital.
Fears of infection, off duty (home)	27 May 2020, Merida, Mexico: A group of people feared a nurse would spread COVID-19 in the neighbourhood and set her car and house on fire.
Fears of infection, off-duty (public)	25 May 2020, unspecified location, Honduras: Several perpetrators threw stones at a nurse on her way back home while accusing her of spreading COVID-19.
Opposition to public health measures, on duty (facility)	3 March 2021, Bovenkarspel, Netherlands: Several perpetrators placed and detonated a pipe bomb outside a COVID-19 testing station.
Fears of infection, on duty (public)	1 March 2021, Zambezi, Zambia: When health workers delivered the body of a man who likely died from COVID-19 for burial, they were attacked by a mob throwing stones at them.

## Data Availability

All data used in this article are publicly available and can be found in the Supplementary Material.

## Supplementary Materials

The following supporting information can be downloaded at: https://www.mdpi.com/article/10.3390/nursrep13020079/s1, Table S1: List of all attack events (.xlsx), Table S2: Data used for the analysis (.xlsx), Table S3: Correlation coefficients (.docx). Reference [37] is cited in the Supplementary Materials.

## Public Involvement Statement

No public involvement in any aspect of this research.

## Guidelines and Standards Statement

This manuscript was drafted against standard reporting procedures in peace and conflict studies using large-scale, geo-coded conflict event datasets. While relevant standards have been established [22,23], and we followed them throughout the manuscript, no formal guidelines are available according to the EQUATOR Network.

## References

[1] WHO (2020). Keep Health Workers Safe to Keep Patients Safe. https://www.who.int/news/item/17-09-2020-keep-health-workers-safe-to-keep-patients-safe-who.

[2] McKay D., Heisler M., Mishori R., Catton H., Kloiber O. (2020). Attacks against health-care personnel must stop, especially as the world fights COVID-19. Lancet.

[3] Devnani M. (2021). COVID-19 and laws for workplace violence in healthcare. BMJ.

[4] Zipf A.L., Polifroni E.C., Beck C.T. (2022). The experience of the nurse during the COVID-19 pandemic: A global meta-synthesis in the year of the nurse. J. Nurs. Scholarsh..

[5] ACLED (2022). Armed Conflict Location & Event Data Project. www.acleddata.com.

[6] Insecurity Insight (2022). Attacked and Threatened: Health Care at Risk. https://map.insecurityinsight.org/health.

[7] Liu J., Gan Y., Jiang H., Li L., Dwyer R., Lu K., Yan S., Sampson O., Xu H., Wang C. (2019). Prevalence of workplace violence against healthcare workers: A systematic review and meta-analysis. Occup. Environ. Med..

[8] Spector P.E., Zhou Z.E., Che X.X. (2014). Nurse exposure to physical and nonphysical violence, bullying, and sexual harassment: A quantitative review. Int. J. Nurs. Stud..

[9] Maguire B.J., Browne M., O’Neill B.J., Dealy M.T., Clare D., O’Meara P. (2018). International survey of violence Against EMS personnel: Physical violence report. Prehospital Disaster Med..

[10] Yang S.Z., Wu D., Wang N., Hesketh T., Sun K.S., Li L., Zhou X. (2019). Workplace violence and its aftermath in China’s health sector: Implications from a cross-sectional survey across three tiers of the health system. BMJ Open.

[11] Franz S., Zeh A., Schablon A., Kuhnert S., Nienhaus A. (2010). Aggression and violence against health care workers in Germany—A cross sectional retrospective survey. BMC Health Serv. Res..

[12] Shiraz S., Lubna Ansari B., Ibrahim H., Mirwais K., Seemin J., Muhammad Naseem K., Munir Akhtar S., Komal Z., Sumera E., Iram Y. (2020). The magnitude and determinants of violence against healthcare workers in Pakistan. BMJ Glob. Health.

[13] Larkin H. (2021). Navigating attacks against health care workers in the COVID-19 era. JAMA.

[14] Dafny H.A., Beccaria G. (2020). I do not even tell my partner: Nurses’ perceptions of verbal and physical violence against nurses working in a regional hospital. J. Clin. Nurs..

[15] Bou-Karroum L., El-Harakeh A., Kassamany I., Ismail H., El Arnaout N., Charide R., Madi F., Jamali S., Martineau T., El-Jardali F. (2020). Health care workers in conflict and post-conflict settings: Systematic mapping of the evidence. PLoS ONE.

[16] Carmichael J.-L., Karamouzian M. (2014). Deadly professions: Violent attacks against aid-workers and the health implications for local populations. Int. J. Health Policy Manag..

[17] Dye T.D., Alcantara L., Siddiqi S., Barbosu M., Sharma S., Panko T., Pressman E. (2020). Risk of COVID-19-related bullying, harassment and stigma among healthcare workers: An analytical cross-sectional global study. BMJ Open.

[18] Bhatti O.A., Rauf H., Aziz N., Martins R.S., Khan J.A. (2021). Violence against healthcare workers during the COVID-19 pandemic: A review of incidents from a lower-middle-income country. Ann. Glob. Health.

[19] Taylor L. (2020). Covid-19 misinformation sparks threats and violence against doctors in Latin America. BMJ.

[20] Devi S. (2020). COVID-19 exacerbates violence against health workers. Lancet.

[21] Ni M.Y., Kim Y., McDowell I., Wong S., Qiu H., Wong I.O.L., Galea S., Leung G.M. (2020). Mental health during and after protests, riots and revolutions: A systematic review. Aust. N. Z. J. Psychiatry.

[22] Eck K. (2012). In data we trust? A comparison of UCDP GED and ACLED conflict event datasets. Coop. Confl..

[23] Raleigh C., Linke A., Hegre H., Karlsen J. (2010). Introducing ACLED: An armed conflict location and event dataset. J. Peace Res..

[24] Gillespie M., O’Loughlin B. (2009). News, media threats and insecurities. Camb. Rev. Int. Aff..

[25] World Bank (2022). World Bank Open Data. https://data.worldbank.org/.

[26] UNDP (2020). Human Development Index (HDI). http://hdr.undp.org/en/content/human-development-index-hdi.

[27] Marshall M.G., Jaggers K., Gurr T.R. (2020). Polity V Annual Time-Series, 1800–2018. http://www.systemicpeace.org/inscrdata.html.

[28] Ritchie H., Ortiz-Ospina E., Beltekian D., Mathieu E., Hasell J., Macdonald B., Giattino C., Roser M. (2022). Coronavirus Pandemic (COVID-19). https://ourworldindata.org/coronavirus.

[29] Nelson R. (2014). Tackling violence against health-care workers. Lancet.

[30] Vento S., Cainelli F., Vallone A. (2020). Violence against healthcare workers: A worldwide phenomenon with serious consequences. Front. Public Health.

[31] Cohn S.K. (2017). Cholera revolts: A class struggle we may not like. Soc. Hist..

[32] Semple K. (2020). ‘Afraid to Be a Nurse’: Health Workers under Attack. https://www.nytimes.com/2020/04/27/world/americas/coronavirus-health-workers-attacked.html.

[33] Tsao S.-F., Chen H., Tisseverasinghe T., Yang Y., Li L., Butt Z.A. (2021). What social media told us in the time of COVID-19: A scoping review. Lancet Digit. Health.

[34] Aharon A.A., Warshawski S., Itzhaki M. (2020). Public knowledge, attitudes, and intention to act violently, with regard to violence directed at health care staff. Nurs. Outlook.

[35] Bollyky T.J., Templin T., Cohen M., Schoder D., Dieleman J.L., Wigley S. (2019). The relationships between democratic experience, adult health, and cause-specific mortality in 170 countries between 1980 and 2016: An observational analysis. Lancet.

[36] Fields L., Perkiss S., Dean B.A., Moroney T. (2021). Nursing and the Sustainable Development Goals: A scoping review. J. Nurs. Scholarsh..

[37] Achen C.H. (2005). Let’s Put Garbage-Can Regressions and Garbage-Can Probits Where They Belong. Confl. Manag. Peace Sci..

