# Supplementary material for: Collective Violence against Health Workers in the Context of the COVID-19 Pandemic
Source: Nurs Rep. 2023 Jun 14;13(2):902–12. doi: 10.3390/nursrep13020079 (PMC10303091; doi:10.3390/nursrep13020079)
Supplement: Supplementary file 1 [file nursrep-13-00079-s001.zip › nursrep-2393431-S3 - Correlation coefficients.pdf]

S3: Correlation coefficients

Correlations between attack risk and socio-economic characteristics at the country level:.

V1	V2	N	Coefficient size (R)	P-Value
Attacks/Population	Physicians/1000	162	-0.0728	0.3559
Attacks/Population	HDI	163	-0.0875	0.2750
Attacks/Population	polity2	163	0.1096	0.1622
Attacks/Population	vaccination rate	69	-0.1105	0.3682

As multivariate regression models have a high risk of model overfit when correlating socio-economic with conflict data [37] for relatively small numbers of cases (here: n = 164), we calculated bivariate Pearson's correlation coefficients.
